# Development of an augmented reality guidance system for head and neck cancer resection

**DOI:** 10.1049/htl2.12062

**Published:** 2023-12-07

**Authors:** Guansen Tong, Jiayi Xu, Michael Pfister, Jumanh Atoum, Kavita Prasad, Alexis Miller, Michael Topf, Jie Ying Wu

**Affiliations:** ^1^ Computer Science Department Vanderbilt University Nashville Tennessee USA; ^2^ Vanderbilt University Medical Center Nashville Tennessee USA

**Keywords:** augmented reality, surgery

## Abstract

The use of head‐mounted augmented reality (AR) for surgeries has grown rapidly in recent years. AR aids in intraoperative surgical navigation through overlaying three‐dimensional (3D) holographic reconstructions of medical data. However, performing AR surgeries on complex areas such as the head and neck region poses challenges in terms of accuracy and speed. This study explores the feasibility of an AR guidance system for resections of positive tumour margins in a cadaveric specimen. The authors present an intraoperative solution that enables surgeons to upload and visualize holographic reconstructions of resected cadaver tissues. The solution involves using a 3D scanner to capture detailed scans of the resected tissue, which are subsequently uploaded into our software. The software converts the scans of resected tissues into specimen holograms that are viewable through a head‐mounted AR display. By re‐aligning these holograms with cadavers with gestures or voice commands, surgeons can navigate the head and neck tumour site. This workflow can run concurrently with frozen section analysis. On average, the authors achieve an uploading time of 2.98 min, visualization time of 1.05 min, and re‐alignment time of 4.39 min, compared to the 20 to 30 min typical for frozen section analysis. The authors achieve a mean re‐alignment error of 3.1 mm. The authors’ software provides a foundation for new research and product development for using AR to navigate complex 3D anatomy in surgery.

## Graphical abstract

This study explores the feasibility of an augmented reality guidance system for resections of positive tumour margins in a cadaveric specimen. The authors present an intraoperative solution that enables surgeons to upload and visualize holographic reconstructions of resected cadaver tissues.

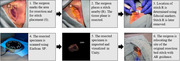

## 1 INTRODUCTION

With an annual incidence of over 660,000 new cases and 325,000 deaths, head and neck cancer stands as the seventh most prevalent cancer worldwide [1]. Surgical intervention is the standard of care for 78% of head and neck cancer patients [2]. Studies have shown surgical margins status to exert vital impact on both local recurrence and overall survival [3]. However, conventional unguided head and neck surgical procedures in the past two decades have yielded unsatisfactory results and thus failed to improve oncologic outcomes [4]. One contributing factor to poor outcomes is the inaccurate relocation of an initially positive margin. Prior studies have shown that relocation error exceeds 10 mm in one‐third of cases [5] and only 20% of re‐resections contain further malignancy [6]. In this work, we propose an augmented reality (AR) guidance system for the relocation of positive margins in head and neck cancer. By integrating three‐dimensional (3D) anatomical data directly into the clinical environment and onto the patient, AR may enhance resection accuracy and improve prognosis. The use of AR in intraoperative navigation and visualization of anatomic structures has shown promise in reducing positive surgical margin rates. In their preclinical study on maxillary cancers, Chan et al. compared the accuracy differences between the ‘ideal’ cutting plan and the AR‐guided virtual osteotomies. They found that positive and close margins are lower for the AR‐assisted osteotomies for maxillary tumours (0.0% vs 1.9% *p* < 0.0001 and 0.8% vs 7.9% *p* < 0.0001) [7]. In the management of sinonasal malignancies, intra‐tumoural cuts were observed in 20.7% unguided simulations versus 9.4% in AR alone scenarios [8].

Our team of engineers and surgeons have worked collaboratively to develop features such as an intuitive interface for uploading 3D scans, voice commands for fine‐tuning manual alignment accuracy, and user‐friendly mesh interaction commands. We evaluate our system on cadavers to demonstrate its feasibility in intraoperative use.

## 2 BACKGROUND

### 2.1 AR‐assisted surgeries

For many malignancies, positive margin rates remain high and subsequently impact patient prognosis [9]. In orthopaedics, this technology has been used for pre‐surgical planning and intraoperative guidance of screw placement [10]. In the neurosurgical field, 3D holograms of preoperative imaging studies have increasingly represented a new modality of surgical navigation [11]. For tissues with high rigidity such as bones, AR has enabled medical professionals to intra‐operatively navigate without the use of fluoroscopy, leading to reduced radiation times [12]. For colorectal cancer, 3D reconstructions of intraoperative ultrasound have been used to achieve adequate resection margins. Ntourakis et al. investigated the impact of AR on the ability to resect missing colorectal liver metastases since modern chemotherapeutics can shrink metastases to the point that they are no longer visible on radiographic imaging. They performed four cases with the registration of the AR‐3D CT model onto the operative image within 6 min and achieved accurate detection with clear resection margins on all cases [13]. In surgical management of lung cancer, Peng et al. found that AR guidance could lead to shorter operating times and less intraoperative blood loss than conventional laparoscopic guidance [14]. Halet et al. applied AR on trans‐thoracic minimally invasive liver resection [15], and Paul et al. presented AR as an educational tool for neurosurgeries [16]. From these studies, we note that deformable anatomical subsites such as the liver and breast present more difficulties than rigid structures such as bones. This difficulty arose from deformable tissue shifting and discrepancies with the pre‐operative scans. Head and neck cancer surgeries often contain multiple subtypes including mucosa, nerves, and grandular structures, posing additional challenges for tumour margin identification [17].

### 2.2 Head and neck re‐resection

If an initial positive margin is encountered in head and neck cancer patients, re‐resection is recommended to achieve complete tumour removal. Following processing and microscopic examination of frozen sections, the results regarding margin status are relayed to the surgeon in the operating room via telephone, often without visual aid. In cases where the margins are found to be close or positive, additional re‐resection is performed prior to the reconstruction phase [17]. Relying solely on the pathologists’ verbal descriptions makes it difficult to relocate the positive margin site and currently, re‐resection does not improve recurrence rates [17]. The head and neck surgeries are still unguided as of 2023 and error‐prone. Prasad et al. performed a review of the margin detection for cT3‐T4 Oral Cavity Squamous Cell Carcinoma. They found no significant decrease in positive margin rates (which remains high at 18.1%) since 2004 [18]. Further, Banoub et al. found that surgeons experience high intraoperative errors when using pre‐operative scans [19]. Specifically, the author concluded that retrospective localization of conventionally labelled margins is not accurate and varies among the care team members [19].

The use of AR has shown to be feasible for head and neck surgeries. Scherl et al. found a mean accuracy of 13 mm when superimposing the holographic model onto the patient's anatomy during parotid tumour surgery [20]. Gsaxner et al. were among the first to introduce the use of HoloLens for immersive visualization of imaging data in head and neck cancer patients [21]. The team's primary goal was to achieve marker‐free image‐to‐phantom registration, and surveys were collected from medical professionals. Rose et al. developed a head‐mounted AR system in Microsoft HoloLens® 2 (Microsoft Corporation) for accurate localization of pathology and anatomical landmarks during open head and neck surgery, using see‐through image projection onto the phantoms [22]. However, the study lacked information regarding the duration required to visualize the model, raising concerns about its feasibility.

Our group previously investigated the feasibility of AR holograms in anatomically aligning 3D‐scanned models of a resection. One study examined the cadaveric specimen of head and neck resection which underwent a 3D scanning process and was subsequently exported to an AR environment using the now‐discontinued Microsoft Mesh application (Microsoft) within the HoloLens 2 [17]. The surgeon manually aligned the holographic representation of the 3D specimen with the resection bed, while recording the accuracy of the alignment and the time intervals throughout the procedure. Uploading to Microsoft Mesh took 2.7 min on average, and the visualization 6.3 min on average.

The current literature on the use of AR during head and neck surgery is still in a preliminary stage, with most research being non‐interventional feasibility studies aiming to determine if AR can enhance the detection of positive margins in different procedures. For example, Necker et al. reported first use of AR to demonstrate the 3D in‐space visualization and manipulation of a virtual resection specimen in mixed reality [23]. Sahovaler et al. reported the first use of AR system in pre‐clinical sinonasal tumour resections [24]. Chan et al. explored the use of AR for image guidance in transoral robotic surgery [25]. Our cadaveric study is a continuation upon current state‐of‐the‐art methods. In previous experiments with cadaver specimens [17], an extended uploading and alignment time was noted. We propose a custom AR guidance system. Further, we introduce a voice‐command method to fine‐tune the alignment margins of the holograms on cadaver specimens.

## 3 METHODS

To overlay digital information onto the real world, we use Microsoft HoloLens 2 as the AR platform. It is a wireless system running on Windows 11 on 4‐GB RAM and 64‐GB storage. The HoloLens application is developed using Unity 3D (Unity 2021.3.1), C# and the Microsoft Mixed Reality Toolkit (MRTK).^1^ In this study, participating surgeons have different post‐ graduation experience levels and no prior exposure to AR. After en bloc resection, the surgical specimen is sent to a 3D scanner to digitally reconstruct its surface topography [26]. The process involves controlling lighting conditions, protecting the scanner turntable, capturing multiple scans, aligning the surface, and exporting the virtual 3D model. This 3D model is then imported into our software, as explained below.

### 3.1 Application design

The software provides surgeons with the ability to customize several key parameters, including distance, size, rotation, motion, and transparency, through either gesture or voice. These parameters aid surgeons in aligning the hologram onto the surgical field in real time.

#### 3.1.1 Uploading and visualization pipeline

**FIGURE 1:**
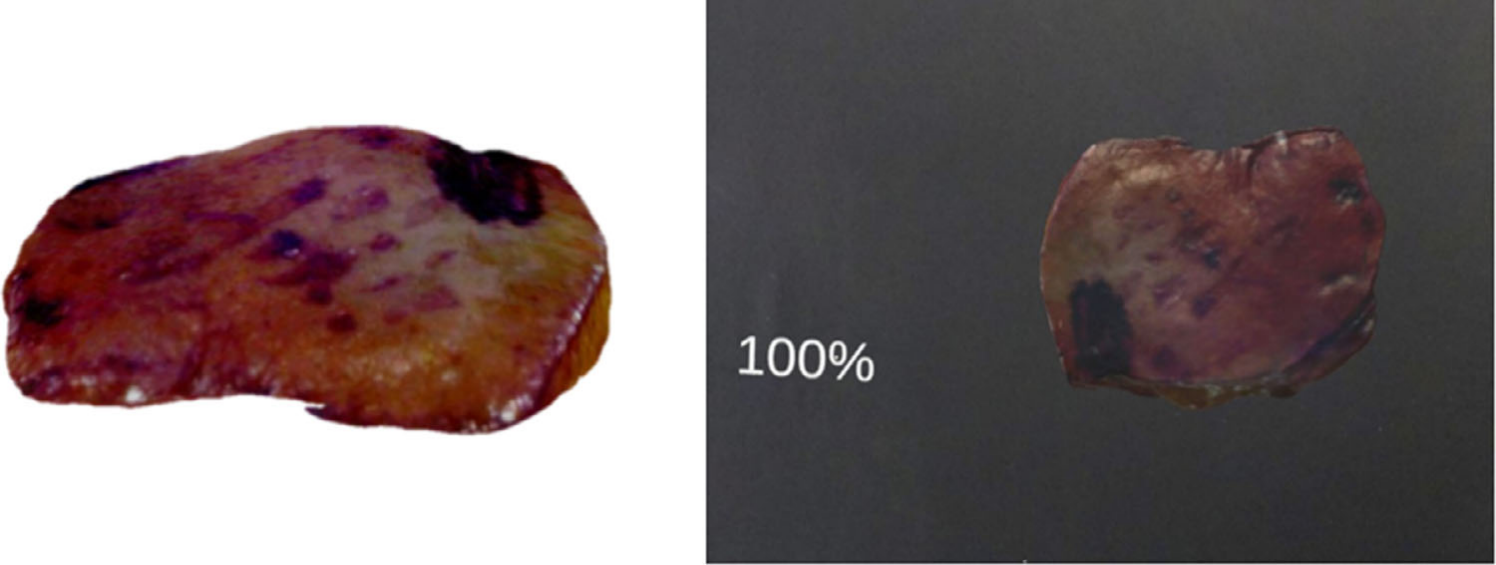
A 3D specimen sample imported in Unity, with the marker (in purple) used to assist surgeons in re‐relocation (Left). Cadaver specimen with its scale displayed in percentage visualized in HoloLens (Right).

**FIGURE 2:**
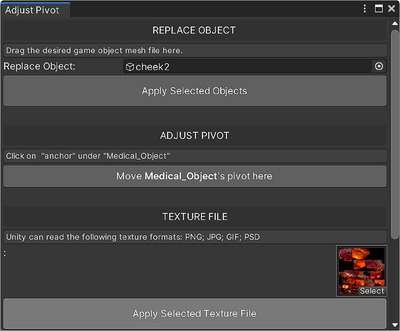
Editor interface for uploading and calibration reduces the learning curve and time in using the software. It consists of three components: uploading the mesh file, uploading the texture file, and resetting the pivot.

In the Unity editor, the pivot point serves as the reference that defines the centre of rotation or scaling for an object. Upon importing a 3D model (see Figure 1, left) into Unity, the pivot point might not align with the object's centre. As a result, users need to manually adjust the pivot point within the Unity editor to accommodate objects of varying shapes and sizes, which slows down visualization. To address this issue, we adopted a pivot adjustment approach.^2^ We have incorporated an editor interface for our software to abstract the complexities of uploading and adjusting 3D files associated with Unity. The editor is straightforward to set up and requires little technical knowledge, alleviating the need to navigate through complex hierarchies in the Unity editor (see Figure 2).

The editor interface consists of three components: uploading the specimen mesh file, uploading the specimen texture file, and resetting the pivot. Each entails a help box with texts for guidance. To upload a mesh file, surgeons drag and drop the .obj file of the target object into the upper box. Once the mesh file successfully uploads, surgeons proceed by selecting ‘Anchor’ child object under the ‘Medical_Object’ and clicking on ‘Move Medical_Object Pivots Here’. This step automatically re‐adjusts the mesh's pivot point, giving it proper positions in relation to the user. Importing the texture file follows a similar process, where surgeons may drop the desired texture file into the lower box shown on the editor interface. After surgeons drag the 3D model into the Unity editor, they click the ‘adjusting pivot’ button on the software's editor interface (see Figure 2). This step triggers the software to realign the object's pivot point to its centre, eliminating the need for repetitive manual adjustments and speeding up visualization. After resetting the pivot point, we position the hologram at 0.5 m from the surgeon. Through this process, our software visually renders the hologram with accurate texture, fixed distance, and scale.

#### 3.1.2 Manipulation with hands

After visualizing the 3D specimen hologram in the HoloLens, surgeons manipulate the object with hand gestures. Specifically, surgeons can pinch and drag the hologram closer or further from the cadaver specimen. We enable far and near interaction using the MRTK 2 package. Accordingly, when the object is positioned between 45 cm and 2 m from the surgeons, a pinch action can be performed with one or two hands and surgeons can use hand rays to grab, scale, or rotate the object [27]. When the object is positioned within 45 cm from the surgeons, they may directly grab and manipulate it.

To improve precision during interaction, we have integrated hand rays into the HoloLens experience. Hand rays are virtual laser‐like beams that extend from the surgeon's hand, giving them a better sense of control and depth perception. Surgeons can use these rays to precisely select and manipulate specific regions of the holographic specimen, making fine adjustments with ease.

#### 3.1.3 Manipulation with voice commands

The HoloLens 2 has device limitations when it comes to manipulating holograms using gestures. One difficulty is hand tracking when dealing with smaller cadaver specimens. Microsoft recommends that interactable hologram meets the minimum size requirements based on its distance from the user. For distances around 45 cm, the recommended size is 1.6 × 1.6 cm. For objects placed at 2 m, the recommended size is 3.5 × 3.5 cm [27]. In our system, the object is located within 50 cm of the users. On average, our resected specimens have lengths ranging from 4.5 to 8 cm and widths ranging from 2 to 5 cm. The precise grabbing, pinching, and manipulation of objects may become more difficult when performing smaller‐scale resections. Cursors and hand rays often fail to align accurately with the objects. Additionally, surgeons’ hands are often occupied for certain tasks, such as placing new stitches in the cancer resection. Thus, they are unable to give clear gesture inputs. For this reason, we implement voice commands in the software as an alternative means of control.

Using voice commands, which differ from hand manipulation where surgeons can only roughly scale the object up or down, they can now make precise size adjustments in increments of 5%. From our initial experiments with cadavers, we determined that 5% is the optimal number for such adjustments. To provide visual feedback on the adjustments made, a progress bar is positioned within the surgeon's field of view, displaying the hologram scale in percentage (see Figure 1, right).

Per surgeons’ needs, we incorporate two additional features that enable a thorough examination of the hologram. To avoid accidental interactions or confusion during complex procedures, the HoloLens employs a contextual awareness system.

Leveraging this system, we make sure that the holographic specimen remains locked in place when it is not being actively manipulated, preventing unintended movements or disruptions during surgery. By issuing voice commands ‘lock’, ‘lock size’, or ‘unlock’, the software enables or disables the near or far interaction (see Table 1). Furthermore, we add voice commands to incrementally adjust the transparency of the hologram by 20%. This capability allows for a gradual modification of transparency, providing a see‐through view on demand.

**TABLE 1:** Application commands.

Input type	Action	Application
Air tap	Pinching of thumb and index finger	Entering the application
Tap, hold, and drag	Holding together thumb and index finger and drag	Placing and adjusting hologram
Both hands tap, hold, and drag	Both hands hold together thumb and index finger and drag	Scaling and rotating hologram
Voice command	Saying ‘initialize’	Creating the hologram
Voice command	Saying ‘bigger’, ‘smaller’, ‘rotate right’, ‘rotate left’, ‘rotate up’, ‘rotate down’	Fine‐tuning alignment margins
Voice command	Saying ‘lock’, ‘lock size’, ‘unlock’, ‘increase transparency’, ‘decrease transparency’	Seeing through specimens
Voice command	Saying ‘back to scale’, ‘back’	Resetting scale and location

## 4 EXPERIMENTAL SETUP

We conducted a cadaveric study to evaluate the feasibility and accuracy of our AR software for visualizing 3D specimens. This study was approved by the Center for Experiential Learning and Assessment at Vanderbilt University Medical Center (VUMC). A total of five simulated oncologic resections were performed, as well as the relocation of the head and neck margin's skin in the resection bed. The study involved the participation of two faculty surgeons and three resident physicians specializing in otolaryngology‐head and neck surgery. None of the surgeons had any previous exposure to AR.

**FIGURE 3:**
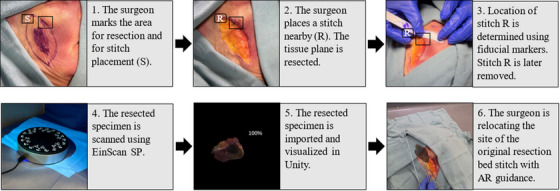
Cadaveric study procedure: this flow chart describes the process of how our software is used in the cadaveric study.

Methods for this relocation experiment were adopted from a previous study [5]. The design of the study is described in detail in Figure 3. Initially, surgeons used a coloured pen to mark the intended area for resection, simulating the process of determining the precise site of the resection bed. Subsequently, they placed one stitch on the specimen and one on the resection bed. Their task was to relocate the stitch on the resection bed after resection. The tissue plane was then resected based on the marked area. Another research team member measured the resection bed stitch location based on the distance to fiducial markers and then removed the stitch. Surgeons were blinded to the measurement process.

In clinical practice, the resected specimen is sent to the pathology lab for microscopic analysis to determine the margin status. This results in the specimen being non‐sterile, making it impossible to place the specimen back to the original resection site to match the margin location. To address this challenge, previous work has proposed using 3D models to guide the realignment process. In this study, we uploaded the 3D model to an AR platform for the surgeon to manipulate and use to guide further resection. The 3D model was obtained through scanning. To ensure scanning accuracy, we chose to scan with EinScan SP (Shining 3D, Hangzhou, China), which provides an accuracy better than 0.05 mm [28]. Its accuracy is below clinically accepted 1 mm measuring error threshold for both the natural head and fixated positions per Hollander et al. [29]. This 3D scanning process generates a Wavefront (.obj) file and an image file that includes the mesh and texture data, which provides visual details and surface characteristics of the object. After the scan, both files were subsequently imported into Unity. After launching the Unity program, surgeons were able to visualize the holograms of the 3D specimen in the HoloLens.

To simulate clinical practice, relocation was not immediately performed after the resection. Instead, surgeons waited for approximately 25 min before conducting the relocation. This process is consistent with normal surgical pathology processing and actual clinical practice. Surgeons relocated the resection bed stitch and placed the new stitch with the guidance provided by the stitch on the holographic specimen model. Another member of the research team used fiducial markers to measure the distance between the original resection bed stitch and the new stitch before calculating the manual realignment error.

## 5 RESULTS

### 5.1 Protocol time measurements

This study included five resections, each resection performed by a different participant (see Table 2), on a single head and neck cadaver as in (Figure 3) were used. All five participants had zero AR experience and varying surgical expertise (shown as post‐graduation years (PGY#)). The greatest dimension of the resection specimens was 5.5 cm (range, 2–5.5 cm). The overall workflow duration fits within the frozen section analysis duration of approximately 25 to 30 min. The frozen section analysis process allows pathologists to rapidly analyze and diagnose tissue samples while the patient is still in the operating room.

**TABLE 2:** Resection performed by participants with varying post‐graduation year (PGY#) experience, showing specimen attributes and realignment error.

Participant years of experience	Anatomic subsite	Specimen size (cm)	Realignment error (mm)
PGY12	Right anterior cheek	5 × 3.5	2
PGY13	Midline scalp	4.5 × 4.5	6
PGY6	Left neck	5 × 2.5	2.5
PGY3	Left preauricular	5.5 × 2	3
PGY6	Lower lip	4.5 × 2.5	2

Abbreviation: PGY, post‐graduation years.

Table 3 shows the time intervals for each step of the intraoperative use. The average time lapsed for the full workflow was 20.51 ± 3.15 min (range, 15.88–23.55 min). 3D scanning time was the only time added to the standard frozen section analysis process. This had an average duration of 10.20 ± 0.52 min (range, 9.35–10.73 min) and accounted for 49.73% of the overall workflow duration. The time lapsed from scanning the specimen to uploading the scanned specimen to Unity constituted about 64.26% of the overall protocol time. Hologram visualization took up 5.12% of the total workflow duration. Hologram realignment took up 21.40%. Refer to Table 3 for a detailed analysis of the time taken for each sub‐process. This demonstrates that the overall protocol takes less time than the frozen section analysis time. Thus, the protocol fits within intraoperative use.

**TABLE 3:** Defining the start and end points for each sub‐process and time intervals.

Interval	Timer start point	Timer stop point	Mean (min)	Standard deviation (min)	Range (min)
3D specimen scanning	Starting 3D scan	Exporting 3D scan	10.20	0.52	9.35–10.73
Importing to unity	Exporting 3D scan	Uploading 3D model	2.98	2.30	1.12–6.97
Hologram visualization	Uploading 3D model	Hologram visible in AR	1.05	0.67	0.35–2.15
Hologram realignment	Hologram visible in AR	Hologram aligned to resection bed	4.39	2.59	0.73–6.90
Full workflow	3D scan initiated	Hologram aligned to resection bed	20.51	3.15	15.88–23.55

Abbreviations: 3D, three‐dimensional; AR, augmented reality.

Note that the full workflow is not the summation of above entries, as frozen section analysis is taken into account. It also includes the stitch placement time.

### 5.2 Realignment accuracy measurements

The mean realignment error was 3.1 mm (range, 2–6 mm) with a standard deviation of 1.67 mm. There was no correlation between the size of the specimen and the realignment error when the virtual specimen was overlaid on the resection region. It is important to recall that every participant performed a singular surgical task. Each participant had a different specimen. The midline scalp specimen showed the highest realignment error of 6 mm when compared to the rest of the specimens.

## 6 DISCUSSION

### 6.1 Workflow duration and realignment accuracy

This cadaveric study reports a mean error of 3.1 mm for manually realigning a 3D holographic specimen back to the cadaveric bed. The size of the specimen does not exhibit any correlation with either the realignment error or the time required to realign the 3D‐scanned specimen back into the surgical defect. The average time for the AR resection protocol is 20.51 ± 3.15 min. The 3D scanning acquisition time adds on average 10.20 ± 0.52 min to the frozen section analysis.

The uploading and visualization steps do not add more time to the frozen section analysis as both run in parallel. Prasad et al. showed that the HoloLens hologram visualization time averages at 6.3 ± 5.7 min [17]. This duration is comparatively shorter than the time required for frozen section analysis. In this study, our tailored user interface and choice of platform reduce the visualization time of scanned specimens. Specifically, the average time to visualize the 3D specimen decreased by 83.33% (from 6.3 ± 5.7 min to 1.05 ± 0.67 min).

Data quality presented to surgeons is enhanced through visualizing 3D specimens [26]. Kerawala and Ong report a mean realignment error of 20 mm while we show a mean of 3.1 mm, a reduction of 84.50% [5]. Upon comparing with our previous cadaver study, we show an improved mean realignment error of 22.50% (from 4 to 3.1 mm) and improved maximum realignment error of 40% (from 10 to 6 mm) [17]. Our study's design takes into account the diversity of surgical cases, ensuring that the realignment error is assessed across different scenarios. This adds robustness to the accuracy results.

In addition, it is noteworthy that there is no correlation between the size of the specimen and the realignment error. It suggests that the accuracy of the virtual specimen overlay is not affected by the physical size of the anatomical structure. This demonstrates that our system's performance remains stable regardless of the complexity or dimensions of the surgical region.

### 6.2 Limitations of the study and future outlook

This study is subject to limitations due to its pre‐clinical nature, tissue shrinkage, and the inherent constraints of the HoloLens 2 device. All results and conclusions are obtained from the cadaveric study.

First, unlike clinical environments, this study is conducted with the close guidance of the software developers. During the relocation process, developers reminded surgeons of correct voice commands and provided instructions on manipulating holograms. In a clinical setting, surgeons need to autonomously navigate the software and AR headset. Therefore, future studies should consider training duration for usability study.

Second, the experimental setup is more distraction‐free than the clinical scenario. Studies reveal that errors in surgery often arise from risk factors such as distractions, interactions between individuals, and reliance on memory [30]. In the cadaveric study, the relocation process is carried out without such factors. In addition, surgeons have no time constraints. Consequently, the accuracy results do not provide conclusive evidence regarding the feasibility in a complex clinical environment.

Third, depending on the type of tissue, there are concerns regarding the potential deformation of both the tumour bed and the specimen during the surgical procedure, with comparatively lesser emphasis placed on the pathological processing. For example, mucosa has been shown to shrink by as much as 10% to 50% during surgery [31]. While we are able to manipulate the size of the 3D specimen hologram using both hand gestures and voice commands, we assume a normal and uniform shrinkage. Our future studies will concentrate on addressing non‐uniform soft‐tissue deformations.

Last, the design of current AR devices creates feasibility difficulties for implementation in a clinical setting. Microsoft's guidance for immersive devices suggests an ideal focal distance of 1.25 to 2.5 m [32]. In clinical environments, objects of interest typically situate at distances between 0.4 and 1.5 m [21]. In our study, the specimen is positioned at approximately 0.5 m from the participant. In addition, while participants have not expressed complaints, it is important to acknowledge that the extended wear time of the headset may lead to comfort issues due to its bulkiness. We envision that future AR surgeries may include head‐mounted displays that integrate with surgical loupes.

In future studies, we plan to assess the feasibility and accuracy of the system in clinical trials. To reduce the burden on clinicians to learn the AR interface, we will explore using Quick Reference (QR) codes as markers for registration and tracking, which allow for more rapid visualization of 3D specimens on the HoloLens platform. The holograms can be automatically aligned and accurately superimposed onto positions of interest.

## 7 CONCLUSION

In our study, we show the feasibility of an AR guidance system for guiding head and neck tumour resection surgeries. Notably, the software's graphical interface and voice command features contribute to usability and compatibility in intraoperative use. Despite its limitations, the software enhances both the efficiency (speed) and accuracy compared to our previous cadaveric study [17]. It reduces the mean visualization time by 83.33% (from 6.3 ± 5.7 min to 1.05 ± 0.67 min). For accuracy, we improve the mean realignment error by 22.50% (from 4 to 3.1 mm) and the maximum realignment error by 40% (from 10 to 6 mm). While our study targets head and neck tumours as a significant use case, the implementation of a precise guidance system for positive margin relocation has the potential to reduce cancer recurrence and enhance clinical outcomes across all surgical cancer resections.

## AUTHOR CONTRIBUTIONS

The project was designed by Michael Topf, Jie Ying Wu, and Guansen Tong. Jiayi Xu, Jumanh Atoum, Michael Topf, Jie Ying Wu conducted the cadaveric study. Guansen Tong and Jiayi Xu curated and analyzed data, conducted formal analyses, carried out investigations, developed methodologies, administered the project, managed resources, designed the software, validated findings, visualized the data, and contributed to both the original draft and the review and editing of the manuscript. Michael Pfister provided valuable resources. Jumanh Atoum participated in data curation and investigation and contributed to the drafting and editing of the manuscript. Kavita Prasad contributed to data curation, methodology, and resource management. Alexis Miller investigated the study. Michael Topf and Jie Ying Wu secured funding, conducted investigations, supervised the project, provided resources, offered supervision, and contributed to the review and editing of the manuscript.

## CONFLICT OF INTEREST STATEMENT

The authors declare no conflict of interest.

## Data Availability

The data that support the findings of this study are available from the corresponding author upon reasonable request.

## Footnotes

1
https://learn.microsoft.com/en‐us/windows/mixed‐reality/mrtk‐unity/mrtk2/packages/mrtk‐packages?view-mrtkunity-2022-05

2
https://github.com/yasirkula/UnityAdjustPivot
